# Tobacco Use among University Students of Jazan Region: Gender Differences and Associated Factors

**DOI:** 10.1155/2014/279231

**Published:** 2014-05-12

**Authors:** Mohamed Salih Mahfouz, Rashad Mohammed Alsanosy, Abdelrahim Mutwakel Gaffar, Anwar Makeen

**Affiliations:** ^1^Department of Family and Community Medicine, Faculty of Medicine, Jazan University, Jazan 82621-7413, Saudi Arabia; ^2^Substance Abuse Research Center (SARC), Jazan University, Jazan, Saudi Arabia; ^3^Medical Research Centre (MRC), Jazan University, Jazan, Saudi Arabia

## Abstract

*Objectives*. The main objectives of this study were to estimate the prevalence of tobacco use and behavioral patterns among undergraduate students at higher education institutions in the Jazan Region of Saudi Arabia during the 2011-2012 academic year and to investigate factors that contribute to tobacco use by gender. *Methods*. A cross-sectional study of 4100 undergraduate students was conducted. An anonymous self-administered questionnaire was used to collect information on the extent and pattern of tobacco consumption and factors associated with tobacco use. *Results*. Current smokers accounted for 16.8% (95% CI: 15.6–18.1) of the population sampled; 25.6% (95% CI: 23.8–27.5) of males were current smokers, whereas only 4.6% (95% CI: 3.6–5.8) of females were current smokers (*P* = 0.000). Multivariate analysis revealed that the most important variables explaining tobacco use among study participants were the use of khat (OR = 14.05; *P* = 0.000), smoking status of friends (OR = 2.25; *P* = 0.000), and substance use by friends (OR = 1.81; *P* = 0.001). *Conclusions*. The results demonstrated that khat use was the main predictor for smoking in Jazan for both males and females. Interventions should be designed to reduce the use of tobacco among university students.

## 1. Introduction 

Tobacco has long been associated with several health problems and is considered a chief preventable risk factor for six of the eight leading causes of morbidity and mortality globally [1]. The World Health Organization (WHO) attributes approximately four million deaths annually to tobacco consumption, and this estimate is expected to increase to eight million by the year 2030 [2]. Moreover, increasing evidence from clinical research suggests that quitting smoking lowers the likelihood of smoking-related diseases and hence increases life expectancy [3, 4].

University students constitute a high risk group regarding the adoption of risky behavior, such as smoking and illicit substance use [5, 6]. University students are prone to risk-taking behavior, which has been associated with the underdevelopment of the orbital-frontal cortex [7]. Additionally, during this stage, identity development is a major concern and youth are susceptible to peer pressure [8].

A compendium of tobacco consumption surveys in Saudi Arabia during the past decade (1999–2009) showed that the prevalence estimates of tobacco use among young adults and university students range from 2.4% to 37.0% [9–13]. A survey was conducted to estimate the prevalence of tobacco use and behavioral patterns among undergraduate students at King Saud University, Riyadh, Saudi Arabia, during the 2008-2009 academic year using a large sample (*n* = 6793) of undergraduate students. The results of this study revealed that the prevalence of smoking among students was 14.5% [10].

Few studies exploring tobacco use patterns among university students have been conducted in the Jazan Region. The objectives of this study were to estimate the prevalence of tobacco consumption and behavioral patterns among undergraduate students at higher education institutions of the Jazan Region of Saudi Arabia during the 2011-2012 academic year and to investigate factors that may contribute to tobacco use among university students by gender.

## 2. Materials and Methods

### 2.1. Design and Study Participants

This observational cross-sectional study targeted students in the three higher education institutions of the Jazan Region: Jazan University, Technical College, and the Academic Health College. The Jazan Region is one of the thirteen regions of the Kingdom of Saudi Arabia. The Jazan Region is located on the tropical Red Sea coast in southwestern Saudi Arabia. Jazan covers an area of 11,671 square kilometers and has a total population of 1.5 million. The inclusion criteria for the study sample were full-time student status, enrolment in one of the university's undergraduate programs during the 2011-2012 academic year, and age between 17 and 24 years.

### 2.2. Sampling

A sample size of 4100 students was calculated based on an estimated prevalence of tobacco use in Saudi Arabia of 25% [10], confidence interval of 95%, error not exceeding 2%, design effect of 1.5, and nonresponse rate of 10%. The sample was stratified first according to the three institutions and then by colleges, and, finally, clusters of classes were randomly selected from each stratum. Probability proportional to size sampling (PPS) was used to determine the number of students in the different categories. Further categories were classified according to specialization into health-related fields, arts and humanities, and other scientific colleges.

### 2.3. Data Collection

A modified version of the GYTS questionnaire was used for data collection [14]. The questionnaire was tested in a pilot study of 50 students. Minor phrasing modifications were made on the original questionnaire after the pretest. The self-administered questionnaire covered demographic data (age, sex, class, and family background, including paternal and maternal education levels, employment, and income levels), tobacco use patterns (type of smoking, frequency, age of initiation, and duration), and possible risk factors for smoking.

### 2.4. Measures

We applied the standard of GYTS for calculating the prevalence of tobacco use [14]. Our measures of tobacco smoking were derived from three questions resulting in two measures: ever smoker and current smoker. A smoker was defined as someone who was currently using ≥1 tobacco product (cigarettes, water-pipes, etc.). Current smoking included daily, nondaily, and occasional smoking in the past 30 days preceding the survey. Ever smoker refers to a person who smoked at least 1 tobacco product (cigarettes, water-pipes, etc.) at any time. Also we asked whether participants ever used khat or any type of substance.

### 2.5. Data Quality and Analysis

To ensure data quality, field work supervisors reviewed the submitted questionnaires daily and any errors or inconsistencies were reviewed immediately. Data entry took place at the Jazan Substance Abuse Research Centre (SARC) under the supervision of a data analysis specialist. Data were entered into Epi Info version 6 and then transferred to SPSS version 17 (SPSS Inc., Chicago, IL, USA). Data analysis involved descriptive statistics as well as inferential statistics. Descriptive statistics included simple tabulation, frequencies, and proportions for categorical variables including cross tabulations. Differences between proportions were assessed for significance using the chi-square test. Variables that were found to be significantly associated with tobacco use were further analyzed using logistic regression models. Multivariate logistic regression analyses were used to identify factors associated with tobacco use by controlling the effects of potential confounding variables. Tobacco use was set as the dependent variable; predictor variables included college category, age of the student, the student's khat use status, friends' smoking status, friends' khat use status, family members who smoke, and friends who use any type of substances. Adjusted odds ratios (ORs) and 95% confidence intervals were reported. Three multivariate models were evaluated for males, females, and all students. Hosmer-Lemeshow statistics were used to evaluate the goodness of the fit of the model. All statistical tests were conducted using a significance level of *P* < 0.05.

### 2.6. Ethical Clearance

This study was conducted in accordance with the ethical standards within the political borders of the Kingdom of Saudi Arabia. All of the participants involved in this study have read, understood, and signed a written consent form. This study was approved by the IRB committee of the Substance Abuse Research Center of Jazan University, Kingdom of Saudi Arabia. Authorization was granted from his Excellency the Vice Chancellor of Jazan University and the deans of the respective colleges and faculties. During distribution of the questionnaire, students were informed that the information collected would be kept anonymous and participation was totally voluntary.

## 3. Results

The overall response rate for distributed questionnaires was 91.80% (3764 out of the target of 4100 students). The mean, median, and mode of student ages were 20.8, 21.0, and 20.0 years, respectively (SD = 1.5), which indicates a normal distribution of the participants ages. As shown in Table 1, 57.5% of students were male and 42.5% were female. Most of the students in the sample were single (86.5%). One-quarter of the students were from health-related colleges (24.7%), 36.7% were in arts and humanities, and 38.6% were in other science colleges, and this distribution represents the actual distribution of students in higher education institutions in Jazan. Health-related colleges included medicine, dentistry, pharmacy, and applied medical sciences. Other science colleges included science, scientific specializations within education, and computer science. Arts and humanities included arts, education, language and translation, administrative sciences, and Sharia and law. Most students (58.8%) were from urban areas and the remaining 41.2% were from rural areas (Table 1). The results indicated that 38.8% of the mothers and 14.8% of the fathers were reported to be illiterate, with only 11.6% of the mothers and 27.1% of the fathers with university degrees or above (Table 1).

As shown in Table 2, the prevalence of ever smoking among students was 21.0% (95% CI: 19.7–22.3) and was significantly higher for males (31.4%, 95% CI: 29.4–33.4) than for females (6.2%; 95% CI: 5.1–7.6) (*P* < 0.001). Current smokers accounted for 16.8% (95% CI: 15.6–18.1) of the population sampled; 25.6% (95% CI: 23.8–27.5) of males were current smokers, whereas only 4.6% (95% CI: 3.6–5.8) of females were current smokers (*P* < 0.000). Cigarette smoking was reported by 11.4% of participants (95% CI: 10.3–12.5) and was significantly higher among males (18.7%; 95% CI: 15.6–18.1) compared to females (2.2%; 95% CI: 1.5–3.1). Water pipe smoking was reported by 7.7% (95% CI: 6.8–8.7) of participants.

Table 3 shows some characteristics of the smokers in this study according to gender. The findings show that 58.8% of the smokers started smoking between the ages of 15–19, with no significant difference between the two sexes (*P* = 0.098). In addition, 46.9% of the students reported that they consume only one cigarette per day, with significant differences between males and females (*P* = 0.005). The majority of water pipe smokers (62.2%) reported that they consumed one rock daily, with significant differences between males and females (*P* = 0.037).

Table 4 compares tobacco use by males and females, according to selected characteristics. The table shows that the prevalence of tobacco smoking increased with increasing age for both male and female students. The prevalence of tobacco use was significantly higher for both males and females who had friends who smoked, friends who chewed khat, and family members who used tobacco. For both genders, the prevalence of smoking was significantly higher for those who reported depression and stress and khat use. An important finding is the couse of khat and tobacco because the findings showed that 79.9% of males and 40% of females use both tobacco and khat.

Table 5 shows the results of logistic regression including statistically significant variables from the univariate analyses. The table depicts the results of three multiple logistic regression models: males, females, and all students. The most important independent predictors of smoking among the male students were student's khat use status (OR = 12.44; *P* = 0.000), friends' smoking status (OR = 1.76; *P* = 0.002), family members who smoke (OR = 1.36; *P* = 0.023), and friends' substance use (OR = 1.77; *P* = 0.002). For females, the most important predictors of tobacco use were student's khat use status (OR = 35.90; *P* = 0.000), friends' smoking status (OR = 3.9; *P* = 0.000) and friends using khat (OR = 2.93; *P* = 0.006). The results for the model of all students suggest that the most important variables explaining tobacco use among study participants were student's khat use status (OR = 14.05; *P* = 0.000), friends' smoking status (OR = 2.25; *P* = 0.000), and friends' substance use status (OR = 1.81; *P* = 0.001). The analysis further suggested that college type and students' ages had no impact on smoking status for males, females, or all students.

## 4. Discussion 

The overall prevalence of current tobacco smoking among students in our sample was estimated to be 16.8%. A large-scale survey conducted at King Saud University [10] found a tobacco smoking prevalence of 14.5%, which is in agreement with our estimate. Our results are also similar to the results of a survey conducted at the University of Sharjah, United Arab Emirates, which estimated the overall prevalence of smoking among its students as 15.1% [8]. A recent systematic review of 12 studies conducted at the university level in KSA indicated that the prevalence of tobacco use among students ranged between 2.4% and 37% [15].

Tobacco use among male students in our study was found to be 25.6%, which was significantly higher than tobacco use by females (4.6%). This finding is consistent with the literature at the KSA level [15, 16] and for Arabic countries [8, 17], where culture plays an important role in female behaviors and habits. Our estimate of the smoking prevalence among females is low compared with the estimate from a study conducted in Dammam in 2005, which found a prevalence of 8.6% among 1020 nonmedical female students [11].

In addition, during the present study we found that the age of smoking initiation was between 15 and 20 years for approximately 58.8% of smokers and less than 15 years for 22.3% of smokers. This finding is in agreement with the literature for Egypt [17] and KSA [18] but differs from findings from the USA [19], where 21% of university students started smoking between ages 15–20 years and 41% started smoking after the age of 20 years, reflecting the decreasing trend of smoking among students in Arabic countries.

The study further indicated that more than 45% of students smoke only one cigarette per day; this trend is similar to the findings of studies in the USA, where there is a declining trend in the number of cigarettes smoked by students [20]. The study also found a strong association between male and female smoking status and khat use of students, khat use or smoking of friends, and family members who smoke. The impact of peers on cigarette smoking and substance use has been found elsewhere and is well documented in the findings of some studies in KSA [10, 18] and Arabic countries [8, 17]. Family also has an impact on tobacco use; the smoking habits of family members had a statistically significant effect on the smoking habits of their offspring or siblings. This interpretation is consistent with findings of previous studies [8, 10, 18].

The findings of the logistic regression demonstrated that the strongest predictor of smoking among males and females was khat chewing; students who were khat chewers were fourteen times more likely to be tobacco smokers. The habit of khat chewing is widespread among all segments of the population in the Jazan Region [21–25]; many studies have documented the couse of khat and tobacco smoking in the region [22, 24]. The logistic regression findings also suggested an association between peer pressure (having friends who smoke) and smoking status. Similar findings have been consistently reported from numerous studies conducted in KSA [10] and different countries in the region, including Bahrain [26] and Egypt [17].

The results of this study must be viewed with some caution. The study was based on self-reported responses and thus may be subject to recall bias and underreporting of tobacco use due to social desirability bias. In addition, the study was based on a cross-sectional study design, which is not suitable for establishing trends and causality between tobacco use and potential risk factors. Despite these limitations, this study provides indicators about tobacco use among Jazan University students and provides a baseline for future prevention programs.

## 5. Conclusion and Recommendations

The prevalence of tobacco use among the study sample is considerable, regardless of the health risks associated with tobacco use. The findings of our study reveal that tobacco smoking is tried by students during the early adolescent years and continues throughout the university years. The results also suggest that khat use is the main predictor for tobacco smoking (couse of khat and tobacco) in Jazan for both males and females. The results further suggest that strategies for smoking prevention and cessation intervention programs should be focused on peer pressure among university students. Campaigns also need to be implemented to create awareness among students at the basic education level to reduce the prevalence of the habit in universities and to avoid its unfavorable health consequences.

## Figures and Tables

**Table 1:** Selected background characteristics of the study population.

Characteristics	Males	Females	Total
	*N* (%)	*N* (%)	*N* (%)
Age group (*n* = 3217)			
Less than 20 years	359 (19.2)	255 (19.0)	614 (16.3)
20-21 years	927 (49.5)	679 (50.2)	1606 (42.7)
22-23 years	490 (26.2)	340 (25.3)	830 (22.1)
24 years or older	97 (5.2)	70 (5.2)	167 (4.4)
College (*n* = 3764)			
Health-related	600 (27.7)	331 (20.7)	931 (24.7)
Arts and humanities	546 (25.2)	834 (52.2)	1380 (36.7)
Other scientific	1019 (47.1)	434 (27.1)	1453 (38.6)
Area of residence			
Rural	1017 (47.0)	532 (33.3)	1549 (41.2)
Urban	1148 (53.0)	1067 (66.7)	2215 (58.8)
Marital status (*n* = 3764)			
Married	98 (4.7)	243 (15.6)	341 (9.1)
Single	1967 (94.3)	1288 (82.6)	3255 (86.5)
Divorced	20 (0.0)	24 (1.5)	44 (1.2)
Widowed	1 (0.1)	4 (0.3)	5 (0.1)
Father's education level (*n* = 3764)			
Illiterate	355 (16.8)	202 (12.6)	557 (14.8)
Primary	586 (27.7)	395 (25.3)	981 (26.1)
Intermediate	292 (13.8)	303 (19.4)	595 (15.8)
Secondary	295 (14.0)	226 (14.5)	521 (13.8)
University or higher	584 (27.7)	437 (28.0)	1021 (27.1)
Mother's education level (*n* = 3764)			
Illiterate	890 (41.9)	569 (36.4)	1459 (38.8)
Primary	592 (27.8)	476 (30.5)	1068 (28.4)
Intermediate	226 (10.6)	193 (12.3)	419 (11.1)
Secondary	170 (8.0)	136 (8.7)	306 (8.1)
University or higher	248 (11.7)	189 (12.1)	437 (11.6)

Total	2165 (57.5)	1599 (42.5)	3764 (100)

**Table 2:** Smoking prevalence and smoking patterns among Jazan University students by gender.

Characteristics	Gender				Total		*P* value
	Male		Female			
	*N* (%)	95% CI	*N* (%)	95% CI	*N* (%)	95% CI
Smoking prevalence							
Ever smokers	656 (31.4)	29.4–33.4	92 (6.2)	5.1–7.6	748 (21.0)	19.7–22.3	0.000
Current smokers	524 (25.6)	23.8–27.5	67 (4.6)	3.6–5.8	591 (16.8)	15.6–18.1	0.000
Type of tobacco use							
Cigarette smokers	336 (18.7)	17.0–20.6	31 (2.2)	1.5–3.1	367 (11.4)	10.3–12.5	0.000
Water pipe smokers	203 (12.1)	10.6–13.8	35 (2.5)	1.8–3.4	238 (7.7)	6.8–8.7	0.000

**Table 3:** Characteristics of smokers.

Characteristics	Males	Females	Total	*P* value
	*N* (%)	*N* (%)	*N* (%)	
Age when starting smoking (*n* = 611)				
Less than 15 years	120 (22.1)	16 (23.5)	136 (22.3)	0.098
15–19 years	326 (60.0)	33 (48.5)	359 (58.8)	
20 years or older	97 (17.9)	19 (27.9)	116 (19.0)	
Number of cigarettes (*n* = 367)				
One	153 (45.5)	19 (61.3)	172 (46.9)	0.005
2–5	60 (17.9)	10 (32.3)	70 (19.1)	
6–9	51 (15.2)	2 (6.5)	53 (14.4)	
10 and more sticks	72 (21.4)	0 (0)	72 (19.6)	
Number of rocks smoked per day (*n* = 238)				
One rock	133 (65.5)	15 (42.9)	148 (62.2)	0.037
2–5 rocks	54 (26.6)	16 (45.7)	70 (29.4)	
6 and more rocks	16 (7.9)	4 (11.4)	20 (8.4)	
Number of snuffs per day (*n* = 74)				
One	31 (49.2)	8 (72.7)	39 (52.7)	0.347
2–5	19 (30.2)	2 (18.2)	21 (28.4)	
6 or more	13 (20.6)	1 (9.1)	14 (18.9)	

**Table 4:** Comparisons of tobacco use for both genders according to selected characteristics.

Variables	Males	Females	*P* value
	*N* (%)	*N* (%)	
Age group			
Less than 20 years	100 (29.2)	6 (2.5)	0.001
20-21 years	260 (28.9)	39 (6.2)	
22-23 years	165 (34.5)	28 (8.8)	
24 years or older	38 (40.0)	5 (8.1)	
Pocket money			
Less than 500 SR	47 (8.7)	26 (35.1)	0.001
501–999 SR	170 (31.4)	18 (24.3)	
1000–2000 SR	268 (49.4)	27 (36.5)	
More than 2000 SR	57 (10.5)	3 (4.1)	
Friends smoke			
Yes	512 (84.8)	44 (53.0)	0.001
No	92 (15.2)	39 (47.0)	
Friends use khat			
Yes	567 (90.4)	39 (43.8)	0.001
No	60 (9.6)	50 (56.2)	
Khat chewing status			
Yes	522 (79.9)	36 (40.0)	0.000
No	131 (20.1)	54 (60.0)	
Family member smokes			
Yes	379 (60.8)	60 (67.4)	0.001
No	244 (39.2)	29 (32.6)	
Feeling depressed			
Yes	404 (68.9)	65 (79.3)	0.050
No	182 (31.1)	17 (20.7)	
Feeling stressed			
Yes	401 (66.3)	689 (81.0)	0.001
No	204 (33.7)	16 (19.0)	

**Table 5:** Multivariate logistic regression analyses for smoking-related factors among study participants.

Category	Males			Females			All students		
	Multivariate analysis			Multivariate analysis			Multivariate analysis		
	OR	95% CI	*P* value	OR	95% CI	*P* value	OR	95% CI	*P*-Value
College type									
Health-related (ref.)	1			1			1		
Arts and humanities	0.78	0.55–1.11	0.172	1.57	0.81–3.04	0.18	0.97	0.71–1.3	0.847
Other scientific	0.76	0.70–1.30	0.764	3.60	1.32–9.79	0.01	1.14	0.86–1.53	0.354
Age group									
Less than 20 years (ref.)	1			1			1		
20-21 years	1.25	0.87–1.71	0.227	0.46	0.15–1.45	0.185	1.10	0.80–1.55	0.546
22-23 years	1.11	0.75–1.66	0.579	0.27	0.08–0.86	0.025	0.92	0.65–1.33	0.690
24 years or older	0.92	0.50–1.70	0.799	0.46	0.09–2.21	0.335	0.86	0.50–1.50	0.597
Student uses khat									
No (ref.)	1						1		
Yes	12.44	9.47–16.34	0.000	35.90	12.6–90.8	0.000	14.05	10.8–18.15	0.000
Friends smoke									
No (ref.)	1								
Yes	1.76	1.24–2.49	0.002	3.90	1.87–8.12	0.000	2.25	1.63–3.09	0.000
Friends use khat									
No (ref.)	1								
Yes	0.83	0.54–1.26	0.383	2.93	1.37–6.27	0.006	1.40	0.98–2.00	0.063
Family member smokes									
No (ref.)	1								
Yes	1.36	1.042–1.77	0.023	1.18	0.63–2.22	0.598	1.27	1.0–1.61	0.054
Substance use by friends									
No (ref.)	1								
Yes	1.77	1.24–2.55	0.002	1.49	0.47–4.75	0.499	1.81	1.27–2.56	0.001
